# Sedation of mechanically ventilated adults in intensive care unit: a network meta-analysis

**DOI:** 10.1038/srep44979

**Published:** 2017-03-21

**Authors:** Zhongheng Zhang, Kun Chen, Hongying Ni, Xiaoling Zhang, Haozhe Fan

**Affiliations:** 1 Department of emergency medicine, Sir Run-Run Shaw Hospital, Zhejiang University School of Medicine, Hangzhou, 310016, China; 2 Department of critical care medicine, Jinhua municipal central hospital, Jinhua hospital of Zhejiang university, Zhejiang, P. R. China

## Abstract

Sedatives are commonly used for mechanically ventilated patients in intensive care units (ICU). However, a variety of sedatives are available and their efficacy and safety have been compared in numerous trials with inconsistent results. To resolve uncertainties regarding usefulness of these sedatives, we performed a systematic review and network meta-analysis. Randomized controlled trials comparing sedatives in mechanically ventilated ICU patients were included. Graph-theoretical methods were employed for network meta-analysis. A total of 51 citations comprising 52 RCTs were included in our analysis. Dexmedetomidine showed shorter MV duration than lorazepam (mean difference (MD): 68.7; 95% CI: 18.2–119.3 hours), midazolam (MD: 10.2; 95% CI: 7.7–12.7 hours) and propofol (MD: 3.4; 95% CI: 0.9–5.9 hours). Compared with dexmedetomidine, midazolam was associated with significantly increased risk of delirium (OR: 2.47; 95% CI: 1.17–5.19). Our study shows that dexmedetomidine has potential benefits in reducing duration of MV and lowering the risk of delirium.

The management of critically ill patients often requires invasive and uncomfortable procedures such as tracheal intubation and physical restraint. Furthermore, the intensive care unit (ICU) environment is filled with noise, which greatly exaggerates the stress and anxiety of conscious patients^1^. There is evidence that stress and anxiety have adverse effects on clinical outcomes, and prevention of exposure to environmental noise can help to improve outcome^2^,^3^. An alternative but important modality is the use of sedatives to prevent critically ill patients from being exposed to hazardous physical and psychological stimulus^4^.

Mechanical ventilation (MV) is among the most commonly used techniques in the ICU. Because of its invasiveness, MV usually brings stressful, uncomfortable and even painful experience to ICU patients^5^,^6^. MV patients are at increased risk of developing delirium^7^. Therefore, international guideline recommends routine use of sedation to fully prevent patients from exposure to these adverse stimuli. However, there is a variety of sedatives that are available for clinical use, including midazolam, dexmedetomidine, propofol and lorazepam^8^. They have different advantages and limitations in clinical use, due to their distinct pharmacological properties. In clinical practice, clinicians usually face with the choice between multiple alternative sedatives. The choice may become difficult when investigators have undertaken head-to-head comparisons of only some of available sedatives. Network meta-analysis allows for simultaneous comparisons of multiple sedatives against each other, providing direct evidence on the choice among multiple sedatives. In this study we performed a systematic review and network meta-analysis of the efficacy and safety of these sedatives, in the hope that it will provide updated and unbiased evidence for clinical practice.

## Methods

### Study population and registration

The study population was critically ill adult patients who required MV. Critically ill patients were defined as those treated in the ICU and were defined as per the original studies. MV included both invasive and non-invasive modes. Pediatric patients (younger than 12 years old) were excluded. Patients included medical patients requiring long-term MV and patients underwent major operation that were transferred to ICU. The study was registered at PROSPERO (CRD42016041920).

### Interventions

Sedatives of any types were deemed suitable for inclusion. However, studies investigating sedation protocol but the types of sedatives are not distinguishable were excluded. Sedatives included midazolam, dexmedetomidine, propofol, clonidine and lorazepam. Studies with one arm involving these sedatives were included. The other arms can be atypical sedatives such as haloperidol, morphine and combination of two types of sedatives. Dexmedetomidine was employed as the base comparator, and other sedatives were compared to it.

### Outcomes

The primary outcome was the duration of MV. Other outcomes included Richmond Agitation Sedation Scale (RASS), Ramsay Sedation Scale (RSS), ICU and hospital length of stay (LOS).

### Search strategy and data extraction

Electronic databases including Pubmed, SCOPUS, ISI web of science, and EMBASE were searched from inception to April 2016. There was no language restriction. Search items included core terms related to critical care, mechanical ventilation, outcomes and sedatives. Examples of searching strategy in PubMed and SCOPUS, as well as the number of extracted citations were provided in supplemental file. Reference lists of relevant articles were screened by hand for potential eligible studies.

Custom-made form was employed to extract data from included trials. Characteristics of studies included the name of the first author, publication year, type of study population, sample size, comparator in each arm and study outcomes. Numerical data on the number of participants in each arm, names of comparators, number of events in each arm, and mean (standard error) were extracted. Two authors (X.Z. and H.F.) independently extracted data, and disagreement was settled by a third opinion (Z.Z.). If the original article reported median and interquartile range, its distribution was assumed to be normal. Data on mean and standard error can be derived according to normal distribution rules. Some continuous outcomes were reported in median and range. They were converted to mean (standard error) according to the equations proposed by Hozo and coauthors^9^.

### Quality assessment of component trials

Only randomized controlled trials were included in our study. Therefore, the qualities of trials were assessed in six aspects: (1) random sequence generation, (2) allocation concealment, (3) blinding of participants and personnel, (4) blinding of outcome assessor (5) incomplete data outcome, (6) selective reporting, and (7) other bias. These items were adapted from the Cochrane Collaboration’s tool for assessing risk of bias^10^.

### Statistical analysis

There were two types of outcome data: continuous and binary outcomes. The former included ICU and hospital LOS, and duration of MV. RASS and RSS were ordinal variables. Binary outcomes included mortality, atrial fibrillation and delirium. Mean difference (MD) was reported for the comparison of continuous outcomes between interventions, and odds ratio (OR) was reported for binary outcomes.

Graph-theoretical methods, which have been routinely applied to electrical networks, were employed for network meta-analysis^11^. Direct comparisons between sedatives were derived from each of the two-arm trials, and were represented by edges in a network plot. Effect sizes from component trials were weighted by the inverse of the observed variance of the treatment effect. For a meta-analytic network, the node corresponds to a treatment strategy, and the edge represents the existing comparisons between treatments. Each two-arm study (e.g. randomized controlled trial) contributes to one comparison. The thickness of the edge is proportional to inverse standard error of random effects model comparing two treatments. Additionally, we employed net heat plot to highlight hot spots of inconsistency between specific direct and indirect evidence in the whole network^12^. The area of a gray square displays the contribution of the direct estimate of one design in the column to a network estimate in a row. The colors are associated with the change in inconsistency between direct and indirect evidence in row design after detaching the effect of column design. Forest plots were employed to show the effect size of each drug, by setting dexmedetomidine as the reference. Effect sizes and corresponding 95% confidence intervals were reported in the forest plots. All statistical analyses were performed using R (version 3.2.3)^13^.

## Results

### Included studies and characteristics

**Figure 1:**
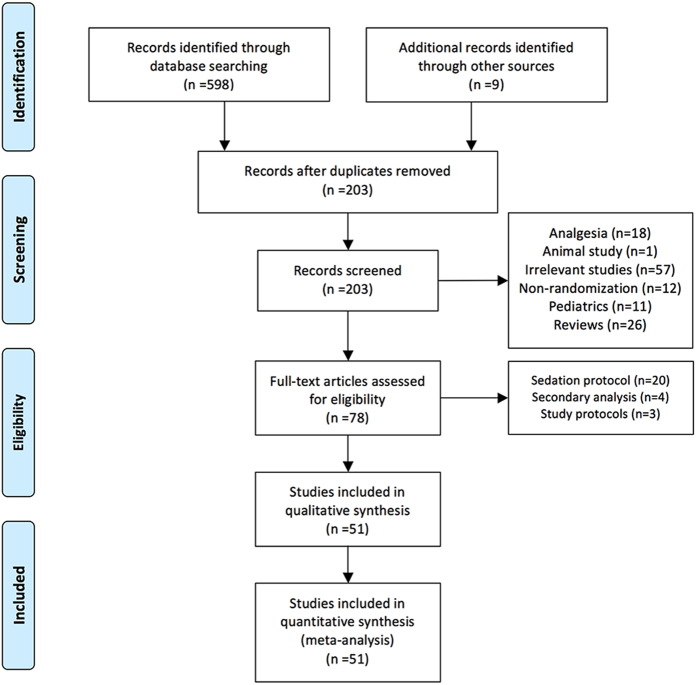
PRISMA flow diagram for study inclusion.

The initial search identified 598 citations. Another 9 studies were added from the references of relevant articles (Fig. 1). After removing duplicates, a total of 203 citations remained for further screening. The titles and abstracts were screened by hand and 125 were excluded because 18 were related to anesthesia, one was animal study, 57 were irrelevant studies, 12 were observational studies, 11 involved pediatric patients, and 26 were reviews. The full-text articles of the remaining 78 citations were screened. Twenty-seven articles were excluded because 20 investigated sedation protocol that the type of sedative drugs could not be identified, 4 studies were secondary analysis of previous reports, and 3 were study protocols. As a result, a total of 51 citations comprising 52 RCTs were included in our analysis^14^,^15^,^16^,^17^,^18^,^19^,^20^,^21^,^22^,^23^,^24^,^25^,^26^,^27^,^28^,^29^,^30^,^31^,^32^,^33^,^34^,^35^,^36^,^37^,^38^,^39^,^40^,^41^,^42^,^43^,^44^,^45^,^46^,^47^,^48^,^49^,^50^,^51^,^52^,^53^,^54^,^55^,^56^,^57^,^58^,^59^,^60^,^61^,^62^,^63^,^64^. The article by Jakob and colleagues comprised two RCTs^38^. Characteristics of component trials are shown in Table 1. These articles were published between the year 1989 and 2016. Study populations included patients underwent major operations requiring ICU admission, and those requiring long-term MV. The sample sizes in included trials ranged from 20 to 500. Most of the trials were two-arm trials, and there were five three-arm trials^20^,^35^,^42^,^63^,^64^. Study endpoints included duration of MV, Richmond Agitation Sedation Scale (RASS), Ramsay Sedation Scale (RSS), ICU and hospital length of stay (LOS). Adverse events included bradycardia, hypotension and death.

### Risk of bias

**Figure 2:**
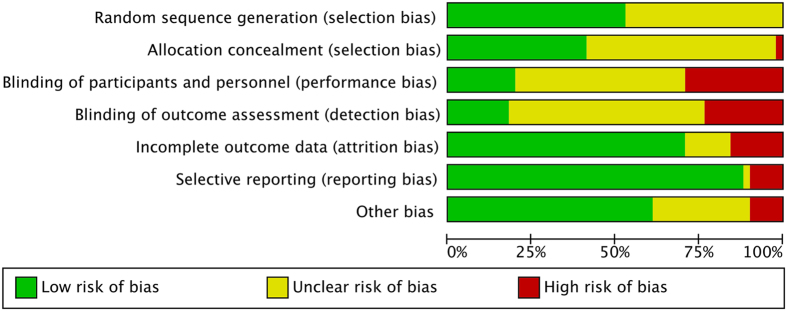
Summary of risk of bias for included trials.

Random sequence generation was adequately described in approximately half of included trials (Fig. 2). In the remaining trials, they did not specifically describe the method of sequence generation. Allocation concealment was properly done in about 21 of the 52 trials. Blinding was difficult to perform because propfol was distinctive in appearance. Attrition bias and reporting bias were generally well performed in included trials. Risk of bias assessment of each trial is present in Supplemental Fig. 1.

**Figure 3:**
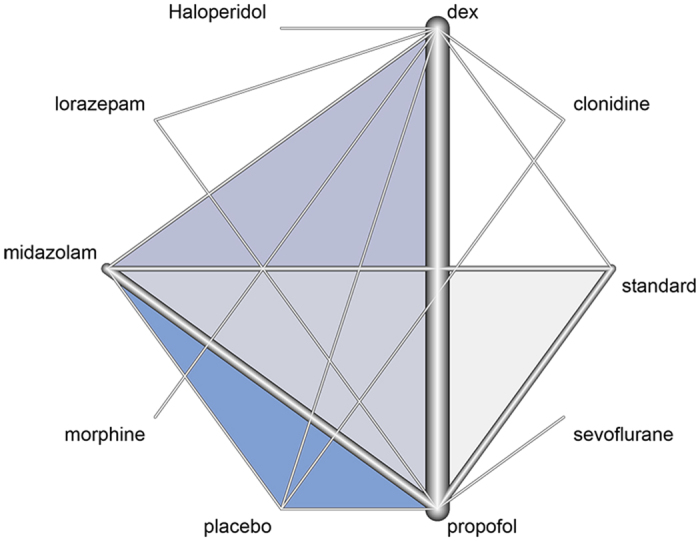
Network of comparators. The nodes in the graph correspond to sedatives and edges display the observed treatment comparisons. The thickness of the edge is proportional to inverse standard error of random effects model comparing two treatments. For example, the dex-propofol comparison appears to be thick, indicating a small standard error for the effect size. Multi-arm studies were highlighted with blue color. For example, there is a study with three arms comprising midazolam, propofol and placebo. The triangle involving these three alternative treatments is filled with blue color.

Network graph for the duration of MV is shown in Fig. 3. Collectively, there were 8 sedatives being compared. They were midazolam, dexmedetomidine, propofol, clonidine, morphine, haloperidol, clonidine and lorazepam. The placebo meant that no sedative was given in that group. Two studies employed propofol and midazolam in combination as the control arm, and we denoted it as the standard^20^,^53^. The thickness of the edge is proportional to inverse standard error of random effects model comparing two treatments. For example, the dex-propofol comparison appears to be thick, indicating a small standard error for the effect size of the comparison (Fig. 3). Multi-arm studies were highlighted with blue color.

### Clinical outcomes

**Figure 4:**
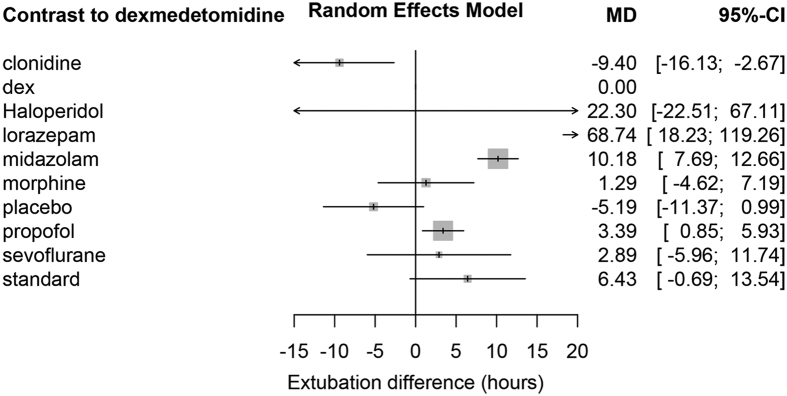
Difference in duration of mechanical ventilation between comparators. Dexmedetomidine was used as the reference.

**Figure 5:**
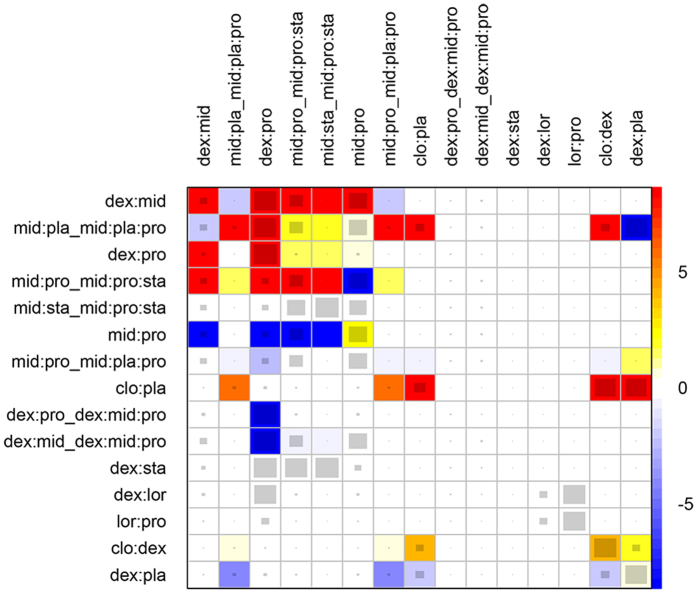
Net heat plot highlights the inconsistency between specific direct and indirect evidence in the whole network. The effect estimates in the row represents pooled effect of direct and indirect effects, and the effects in the column corresponds to the direct effect. There are gray squares in some cells, and the area size is proportional to the contribution of one design in a column that is estimated from direct comparison to an overall network estimate in a row. Also note there are different colors in the cells. After detaching the effect of column design, there will be a change in the inconsistency between direct and indirect estimates. Blue colors indicate an increase in the inconsistency between direct and indirect estimates, and warm colors indicate the opposite (the intensity of the color is proportional to the magnitude of change). The pairwise contrasts corresponding to designs of three-arm studies are marked by the symbol ‘_’ following the treatments of the design.

**Figure 6:**
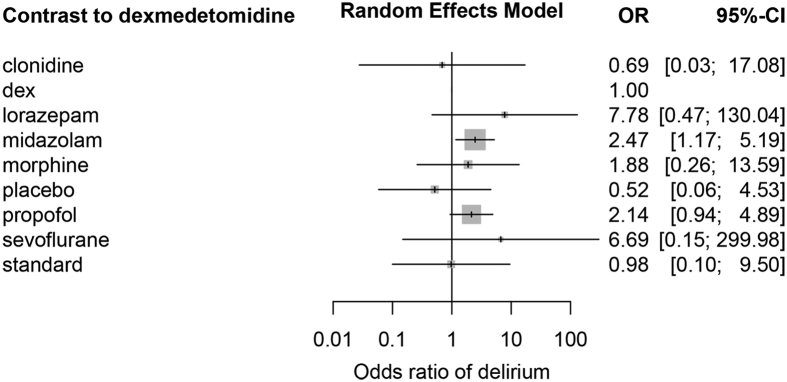
Odds of delirium in each sedative, as compared to that in dexmedetomidine group.

MV duration was reported in most studies. Random effects model was employed to combine the results. Dexmedetomidine showed shorter MV duration than lorazepam (MD: 68.74; 95% CI: 18.2–119.3 hours), midazolam (MD: 10.2; 95% CI: 7.7–12.7 hours) and propofol (MD: 3.4; 95% CI: 0.9–5.9 hours). However, MV duration in dexmedetomidine group was longer than clonidine (MD: −9.4; 95% CI: −16.1–-2.7 hours) and placebo (MD: −5.2 95% CI: −11.4–0.99 hours). There were no significant differences for MV duration in dexmedetomidine group as compared to that in haloperidol, morphine, sevoflurane and standard groups (Fig. 4). There were large changes in inconsistency between direct and indirect evidence in design dex:pro (shown in the row) after detaching the effect of corresponding column design (Fig. 5). As compared to dexmedetomidine, midazolam was associated with significantly increased risk of delirium (OR: 2.47; 95% CI: 1.17–5.19). Propofol was associated with increased risk of delirium with marginal statistical significance (OR: 2.14; 95% CI: 0.94–4.89). There was no difference in the risk of delirium in other comparisons (Fig. 6). There was no difference in the ICU LOS in all comparisons (Suppl. Fig. 2), except that haloperidol was associated with longer ICU stay (MD: 5; 95% CI: 1.8–8.2 days). Dexmedetomidine was associated with shorter LOS in hospital than propofol (MD: 4.6; 95% CI: 1.2–8.1 days, Suppl. Fig. 3). There is no difference in mortality between dexmedetomidine and other sedatives (Suppl. Fig. 4). There were no differences in RSS, RASS or atrial fibrillation between dexmedetomidine and other comparators (Suppl. Figs 5 to 7).

## Discussion

The present study showed that dexmedetomidine was able to reduce MV duration in critically ill patients, as compared to conventional sedatives such as lorazepam, midazolam and propofol. In addition, dexmedetomidine was associated with lower risk of delirium than that of midazolam and propofol. Dexmedetomidine was also associated with shorter hospital LOS than propofol. Propofol showed a shorter MV duration when compared to midazolam, and it has similar risk of delirium to midazolam. There were no significant differences between sedatives in other important outcomes such as mortality, ICU and hospital LOS.

Several meta-analyses of sedatives in critically ill patients have been conducted before our study^65^,^66^,^67^. Fraser’s study included only 6 trials comparing Benzodiazepine versus nonbenzodiazepine-based sedation for the mechanically ventilated patients. Numerous studies in this area have been published since that time, and the evidence needs to be updated. In our study, we included 51 citations that were far more than that included in Fraser’s study. Furthermore, previous studies did not perform meta-analysis in network framework. By using conventional pairwise meta-analysis, many types of sedatives had to be combined as the control group, ignoring the fact that these sedatives were different in their pharmacological properties. In clinical practice, clinicians usually face with the choice between multiple alternative sedatives. The choice may become difficult when investigators have undertaken head-to-head comparisons of only some of available sedatives. Chen’s study highlighted dexmedetomidine, and other sedatives were used as the controls. Cruickshank’s study also had the same limitation. We believe that all other sedatives are different in their efficacy and safety profiles. In this situation, network meta-analysis is more appropriate because it allows for simultaneous comparisons of multiple interventions against each other. With respect to searching database, SCOPUS was not searched in Cruickshank’s study. In the present study, many publications in non-English language were retrieved from SCOPUS. Missing these citations may result in biased estimates of pooled results, also known as publication bias. Although there are significant differences in included trials, some aspects of our findings are consistent with previous meta-analyses. For example, Cruickshank’s study found that dexmedetomidine was effective in reducing time to extubation in the ICU patients, and risk of bradycardia but not of overall mortality is higher among patients treated with dexmedetomidine^67^. However, Fraser’s study found a similar prevalence of delirium between patients treated with benzodiazepine versus nonbenzodiazepine sedatives^65^. This discrepancy can be partly explained by the limited number of component trials and the combination of different types of sedatives as the control group.

Several limitations in our study need to be acknowledged. First, the study population involved critically ill patients that were heterogeneous in nature. Patients after major operation and medical ICU patients requiring long-term MV were enrolled. However, the common feature was that they all needed MV, and sedatives were used for the same purpose for them. Second, there were significant risks of bias in most of enrolled trials. For example, blinding to participants and investigators was not performed due to the appearance of propofol. Also, specific methods to generate random sequence were not explicitly reported in nearly half of the trials. Third, more than two thirds of component trials had a sample size of less than 100, which were typically small studies. As a result, the systematic review may be subject to small study effect bias^68^.

In conclusion, our study showed that dexmedetomidine had potential benefits in reducing duration of MV and lowering the risk of delirium. Propofol is considered superior to midazolam in terms of MV duration. Adverse events of hypotension and bradycardia should be closely monitored when sedatives are used for MV patients.

## Additional Information

**How to cite this article:** Zhang, Z. *et al*. Sedation of mechanically ventilated adults in intensive care unit: a network meta-analysis. *Sci. Rep.* **7**, 44979; doi: 10.1038/srep44979 (2017).

**Publisher's note:** Springer Nature remains neutral with regard to jurisdictional claims in published maps and institutional affiliations.

## Supplementary Material

Supplementary Dataset 1

## Figures and Tables

**Table 1:** Characteristics of included studies.

Studies	Study population or setting	Sample size	Comparators	Outcomes
Abd Aziz^14^	Cardiac surgery	28	DEX; morphine	Sedation scores; pain; HR; ABP; extubation time
Aghdaii^15^	CABG	50	Propofol; Midazolam	BP; HR; extubation time; ICU LOS
Aitkenhead^16^	MV > 12 H	101	Propofol; Midazolam	MV duration; BP; HR; biochemistry profile
Aydogan^17^	Scoliosis surgery	32	DEX; Midazolam	Adverse events; MV duration; ICU LOS
Balkanay^18^	CABG	90	Placebo; DEX	Renal injury; hospital and ICU stay
Barrientos-Vega^19^	MV > 24 h	108	Propofol; Midazolam	Cost; extubation time; mortality;
Carrasco^20^	CABG	75	Propofol; Midazolam; Propofol + Midazolam	HR; BP; extubation time
Carson^21^	Medical ICU; MV > 48 hours	132	Lorazepam; propofol	Ventilator days; 28-day ventilator-free survival, ICU and hospital LOS, and hospital mortality.
Cernaianu^22^	MV	95	Lorazepam; Midazolam	HR; MAP; hemodynamic parameters;
Chamorro^23^	MV > 48 h	98	Propofol; Midazolam	Sedation efficacy; hypotension;
Corbett^24^	CABG	89	DEX; propofol	Perception of ICU experience
Djaiani^25^	Cardiac surgery	183	DEX; propofol	Delirium; extubation time;
Abdulatif^26^	MV	40	DEX; propofol	
Elbaradie^27^	Postoperative MV	60	DEX; propofol	RSS; BIS; extubation time
Eremenko^28^	Cardiac surgery	55	DEX; propofol	Duration of MV; adverse events;
Esmaoglu^29^	Eclampsia	40	Midazolam; DEX	Heart rate, blood pressure, RSS; ICU LOS;
Gupta^30^	Postabdominal surgery MV > 24 h	40	Midazolam; DEX	Heart rate; ICU LOS; duration of MV
Hall^31^	MV	99	Midazolam; propofol	ICU LOS; duration of MV
Hellström^32^	Cardiac surgery	99	Sevoflurane; propofol	ICU and hospital LOS; duration of MV; agitation
Herr^33^	CABG	295	DEX; propofol	Adverse events; extubation time
Higgins^34^	CABG	84	Propofol; midazolam	HR; BP; cardiac output
Hu^35^	ICU MV > 24 h	76	DEX; propofol; midazolam	extubation time; delirium; adverse events;
Huang^36^	Non-invasive MV	62	DEX; midazolam	Delirium; ICU LOS; duration of MV; adverse events
Huang^37^	Major surgery MV	108	DEX; propofol	Adverse events; ICU LOS; duration of MV
Jakob^38^	Prolonged MV	500 + 498	DEX; propofol; midazolam	ICU LOS; RASS; duration of MV
Jalonen^39^	CABG	80	DEX; placebo	Hemodynamics; myocardial function;
Kim^40^	CABG	153	DEX; placebo	ICU and hospital LOS; mortality; cardiac function
MacLaren^41^	Mixed ICU; MV > 12 h	23	DEX; midazolam	Duration of MV; ICU LOS
Maldonado^42^	Cardiac surgery	90	DEX; midazolam; propofol	Duration of MV; ICU and hospital LOS
Memis^43^	Septic shock	40	DEX; propofol	Hemodynamics, ICU LOS; mortality
Pandharipande^44^	Mixed ICU; MV > 120 h	103	DEX; lorazepam	Duration of MV; ICU LOS; mortality; efficacy of sedation
Reade^45^	Delirious MV	20	Haloperidol; DEX	MV duration; adjunct propofol; ICU LOS
Reade^46^	Agitated delirium	71	DEX; placebo	duration of MV; ICU and hospital LOS; mortality
Ren^47^	CABG	162	DEX; placebo	BP; HR; other arrhythmia
Riker^48^	MV > 3 d	366	DEX; midazolam;	duration of MV; ICU LOS; mortality; delirium
Roekaerts^49^	Coronary artery surgery	30	Midazolam; propofol	BP; HR; extubation time;
Rubino^50^	Surgery for type-A aortic dissection	30	Clonidine; placebo	Delirium; duration of MV; ICU LOS;
Ruokonen^64^	MV requiring long-term sedation	85	DEX; midazolam; propofol	duration of MV; ICU LOS; delirium
Sakarya^51^	CABG	40	midazolam; propofol	Adverse events; MV duration
Shah^52^	Postoperative MV	30	DEX; propofol	Safety and efficacy;
Shehabi^53^	MV > 12 h	37	DEX-based EGDS; standard care	ICU and hospital LOS; mortality; delirium; MV duration
Shehabi^54^	Cardiac surgery	299	DEX; morphine	Delirium; ICU and hospital LOS; mortality; adverse events
Song^55^	ICU MV > 3d	90	DEX; midazolam	Delirium; ICU LOS;
Soro^56^	CABG	73	Sevoflurane; propofol	ICU and hospital LOS; mortality; adverse events
Srivastava^57^	MV > 12 h	70	Clonidine; DEX	MV duration; hypotension; bradycardia
Tasdogan^58^	Severe sepsis	40	DEX; propofol	Mortality; IAP; MV duration; ICU LOS
Venn^59^	Postoperative MV > 8 h	20	DEX; propofol	RSS; BIS; MV duration; ICU LOS
Wan^60^	SICU	200	DEX; midazolam	Delirium; MV duration; ICU LOS
Weinbroum^61^	MV	67	Midazolam; propofol	Hypotension; agitation;
Yapici^62^	Cardiac surgery	72	DEX; midazolam	MV duration; RASS;
Zhang^63^	COPD MV > 48 h	162	Midazolam; propofol;control	Vital signs; extubation time;

CABG: coronary artery bypass grafting; DEX: Dexmedetomidine; MV; mechanical ventilation; RSS: Ramsay sedation score; BIS: bispectral index; ICU: intensive care unit; LOS: length of stay; EGDS: early-goal directed sedation; IAP: intra-abdominal pressure; SICU: surgical ICU.
