# Supplementary material for: Sedation of mechanically ventilated adults in intensive care unit: a network meta-analysis
Source: Sci Rep. 2017 Mar 21;7:44979. doi: 10.1038/srep44979 (PMC5359583; doi:10.1038/srep44979)
Supplement: Supplementary Dataset 1 [file srep44979-s1.pdf]

Sedation of mechanically ventilated adults in intensive care unit: a network meta-analysis

Zhongheng ZHANG1; Kun Chen2; Hongying Ni2; Xiaoling Zhang2; Haozhe Fan2

Searhing strategy and reults

Scopus 2016-4-18
(TITLE-ABS-KEY ("dexmedetomidine") OR TITLE-ABS-KEY ("clonidine") OR TITLE-ABS-KEY ("propofol") OR TITLE-ABS-KEY ("midazolam") OR TITLE-ABS-KEY (" Lorazepam ")) AND (TITLE-ABS-KEY ("critical care") OR TITLE-ABS-KEY ("intensive care") OR TITLE-ABS-KEY ("icu")) AND (TITLE-ABS-KEY ("randomization") OR TITLE-ABS-KEY ("randomized") OR TITLE-ABS-KEY ("randomly") OR TITLE-ABS-KEY ("random")) AND (TITLE-ABS-KEY ("mechanical ventilation") OR TITLE-ABS-KEY ("ventilated")) AND (TITLE-ABS-KEY ("mortality") OR TITLE-ABS-KEY ("duration") OR TITLE-ABS-KEY ("length of stay") OR TITLE-ABS-KEY ("delirium")) AND (LIMIT-TO (DOCTYPE , "ar"))

185

pubmed
((((((((mortality[Title/Abstract]) OR duration[Title/Abstract]) OR length of stay[Title/Abstract]) OR delirium[Title/Abstract])) AND ((mechanical ventilation[Title/Abstract]) OR ventilated[Title/Abstract])) AND ((((randomization[Title/Abstract]) OR random[Title/Abstract]) OR randomly[Title/Abstract]) OR randomized[Title/Abstract])) AND (((critical care[Title/Abstract]) OR intensive care[Title/Abstract]) OR icu[Title/Abstract])) AND (((((dexmedetomidine[Title/Abstract]) OR clonidine[Title/Abstract]) OR propofol[Title/Abstract]) OR midazolam[Title/Abstract]) OR Lorazepam[Title/Abstract])

111

Figure 1

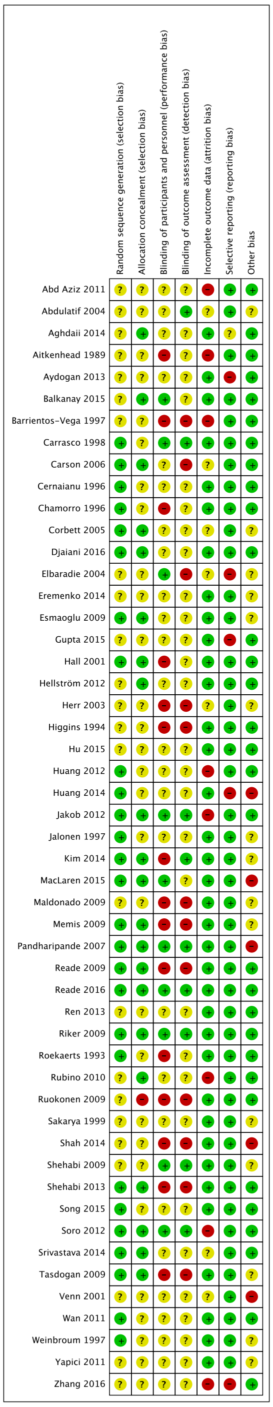
Random sequence generation (selection bias)
Allocation concealment (selection bias)
Blinding of participants and personnel (performance bias)
Blinding of outcome assessment (detection bias)
Incomplete outcome data (attrition bias)
Selective reporting (reporting bias)
Other bias
Abd Aziz 2011
Abdulatif 2004
Aghdaii 2014
Aitkenhead 1989
Aydogan 2013
Balkanay 2015
Barrientos-Vega 1997
Carrasco 1998
Carson 2006
Cernaianu 1996
Chamorro 1996
Corbett 2005
Djaiani 2016
Elbaradie 2004
Eremenko 2014
Esmaoglu 2009
Gupta 2015
Hall 2001
Hellström 2012
Herr 2003
Higgins 1994
Hu 2015
Huang 2012
Huang 2014
Jakob 2012
Jalonen 1997
Kim 2014
MacLaren 2015
Maldonado 2009
Memis 2009
Pandharipande 2007
Reade 2009
Reade 2016
Ren 2013
Riker 2009
Roekaerts 1993
Rubino 2010
Ruokonen 2009
Sakarya 1999
Shah 2014
Shehabi 2009
Shehabi 2013
Song 2015
Soro 2012
Srivastava 2014
Tasdogan 2009
Venn 2001
Wan 2011
Weinbroum 1997
Yapici 2011
Zhang 2016

Figure 2

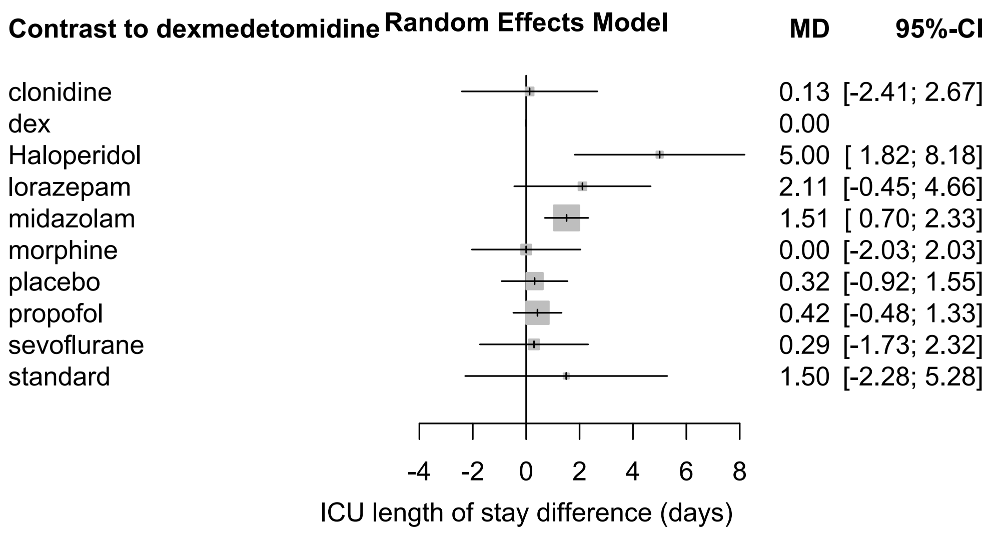

Contrast to dexmedetomidine
Random Effects Model
MD
95%-CI
clonidine
dex
Haloperidol
lorazepam
midazolam
morphine
placebo
propofol
sevoflurane
standard
0.13 [-2.41; 2.67]
0.00
5.00 [1.82; 8.18]
2.11 [-0.45; 4.66]
1.51 [0.70; 2.33]
0.00 [-2.03; 2.03]
0.32 [-0.92; 1.55]
0.42 [-0.48; 1.33]
0.29 [-1.73; 2.32]
1.50 [-2.28; 5.28]
-4
-2
0
2
4
6
8
ICU length of stay difference (days)

Figure 3

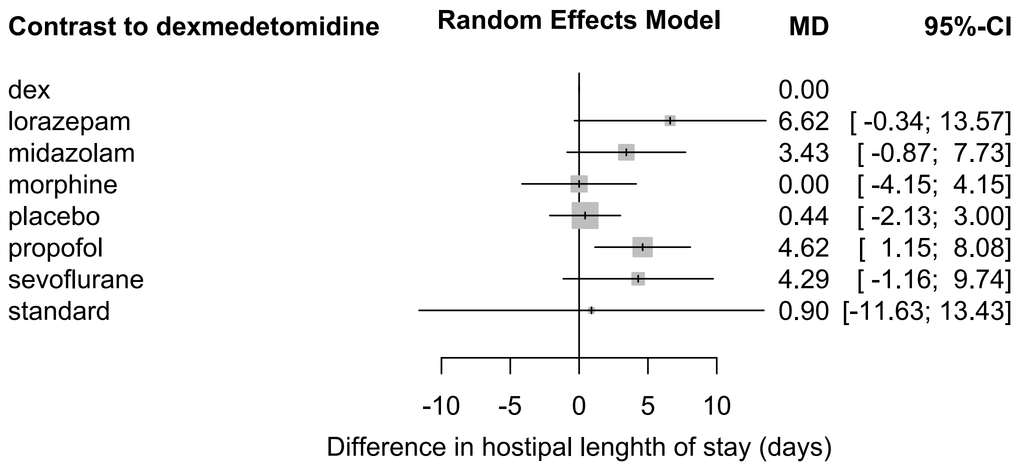
Contrast to dexmedetomidine
Random Effects Model
MD
95%-CI
dex
0.00
lorazepam
6.62 [-0.34; 13.57]
midazolam
3.43 [-0.87; 7.73]
morphine
0.00 [-4.15; 4.15]
placebo
0.44 [-2.13; 3.00]
propofol
4.62 [1.15; 8.08]
sevoflurane
4.29 [-1.16; 9.74]
standard
0.90 [-11.63; 13.43]
-10
-5
0
5
10
Difference in hostipal lenghth of stay (days)

Figure 4

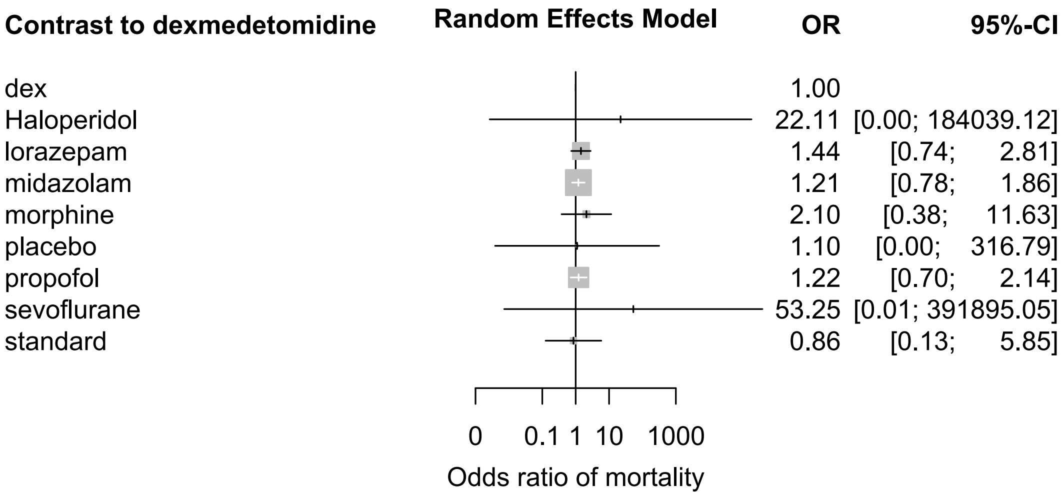
Contrast to dexmedetomidine
Random Effects Model
OR
95%-CI
dex 1.00
Haloperidol 22.11 [0.00; 184039.12]
lorazepam 1.44 [0.74; 2.81]
midazolam 1.21 [0.78; 1.86]
morphine 2.10 [0.38; 11.63]
placebo 1.10 [0.00; 316.79]
propofol 1.22 [0.70; 2.14]
sevoflurane 53.25 [0.01; 391895.05]
standard 0.86 [0.13; 5.85]
0
0.1
1
10
1000
Odds ratio of mortality

Figure 5

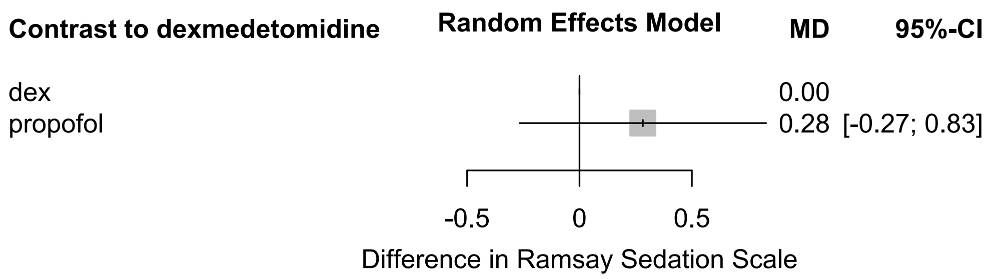
Contrast to dexmedetomidine
Random Effects Model
MD
95%-CI
dex
0.00
propofol
0.28 [-0.27; 0.83]
-0.5
0
0.5
Difference in Ramsay Sedation Scale

Figure 6

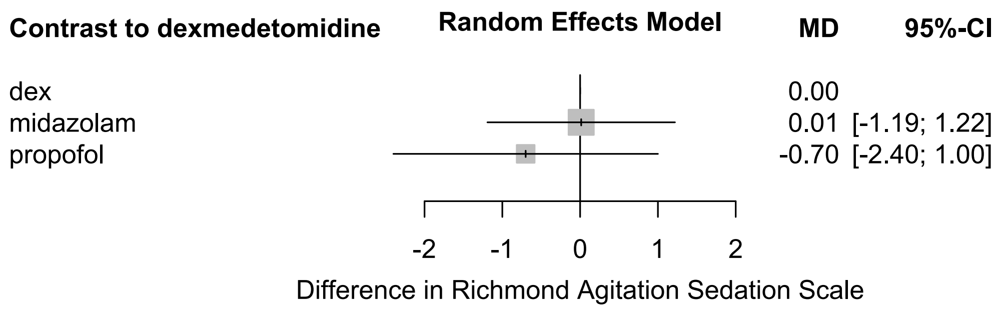

Contrast to dexmedetomidine
Random Effects Model
MD
95%-CI
dex
midazolam
propofol
0.00
0.01 [-1.19; 1.22]
-0.70 [-2.40; 1.00]
-2
-1
0
1
2
Difference in Richmond Agitation Sedation Scale

Figure 7

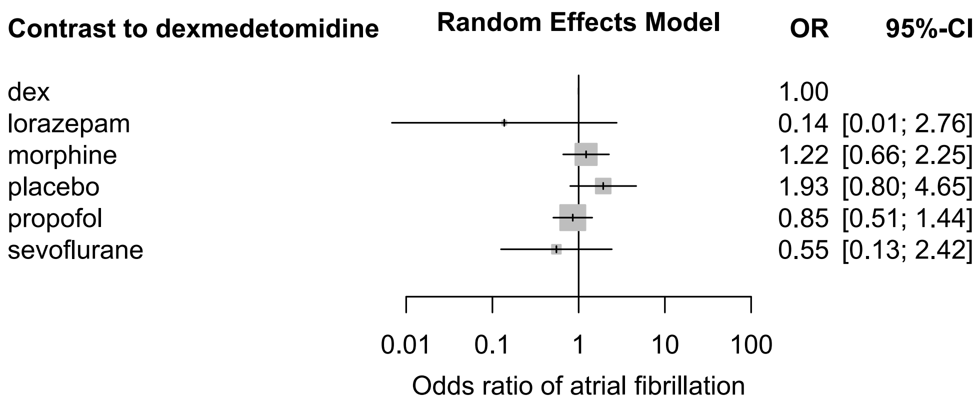
Contrast to dexmedetomidine
Random Effects Model
OR
95%-CI
dex
lorazepam
morphine
placebo
propofol
sevoflurane
1.00
0.14 [0.01; 2.76]
1.22 [0.66; 2.25]
1.93 [0.80; 4.65]
0.85 [0.51; 1.44]
0.55 [0.13; 2.42]
0.01
0.1
1
10
100
Odds ratio of atrial fibrillation
